# The relationship between epicardial adipose tissue and mortality in systemic sclerosis patients: 10-year follow-up results

**DOI:** 10.3389/fcvm.2026.1722600

**Published:** 2026-03-25

**Authors:** Gamze Yeter Arslan, Serdar Söner

**Affiliations:** 1 Department of Cardiology, Kepez State Hospital, Antalya, Türkiye; 2 Department of Cardiology, Gazi Yaşargil Training and Research Hospital, Diyarbakır, Türkiye

**Keywords:** cardiovascular disease, epicardial adipose tissue, mortality, scleroderma (or systemic sclerosis), systemic sclerosis

## Abstract

**Background:**

Systemic sclerosis (SSc) is a connective tissue disease with cardiac involvement characterized by autoimmunity, vascular damage, and fibrosis. Recent studies suggest that epicardial adipose tissue (EAT) may be associated with poor cardiovascular outcomes in patients with SSc and cardiovascular comorbidities. Our aim in this study was to investigate the relationship between epicardial adipose tissue thickness and mortality in patients with systemic sclerosis.

**Methods:**

Sixty patients who presented to cardiology and rheumatology outpatient clinics between January 2013 and December 2015 were retrospectively included in the study. Based on 10-year follow-up results, patients were divided into two groups: those who died and those who survived (30 patients in each group).

**Results:**

In Pearson correlation analyses, we found a positive correlation between EAT and mean pulmonary arterial pressure and right ventricular myocardial performance index, and a negative correlation between tricuspid annular plane systolic excursion and aortic propagation velocity. It was significantly associated with all-cause mortality (*p* < 0.001) and was an independent predictor in multivariable Cox regression analysis (HR = 1.120; 95% CI = 1.050–1.195; *p* < 0.001). In ROC analyses, a cut-off value of 6.05 mm predicted mortality with 80% sensitivity and 70% specificity (AUC = 0.783, *p* < 0.001).

**Conclusions:**

Based on the results of our study, we believe that EAT may predict mortality in SSc patients. Adding EAT, which can be measured noninvasively and with standard echocardiography in SSc patients, to routine assessments may provide clinicians with valuable predictive information for patient management.

## 1 Introduction

Systemic sclerosis (SSc) is a connective tissue disease characterized by autoimmunity, vascular damage, and fibrosis (1). It is a rare condition that has a substantial impact on health, affecting about 2.5 million people globally (2). The 10-year survival rate varies between 66% and 82% in population-based studies (3–5). The pathophysiology of the illness is still complicated and not fully understood (6).

In most cases, myocarditis, fibrosis, pulmonary hypertension, and anomalies of the blood vessels are the primary causes of cardiac involvement (7). Pulmonary arterial hypertension due to SSc is associated with right heart failure and increased risk of MACE, and is a leading cardiovascular cause of death, accounting for 20%–30% of deaths in individuals with SSc (8).

It has been shown that adipose tissue is an endocrine and paracrine organ that produces a range of chemicals involved in immune responses, inflammation, and energy metabolism (9, 10).

Epicardial adipose tissue (EAT) has been shown to predict the risk of major adverse cardiovascular events (MACE), induce microvascular dysfunction and fibrosis, and promote inflammation in adjacent cardiac tissues as well (11, 12). Prior research has demonstrated a correlation between the existence of SSc and the EAT thickness as determined by echocardiography in front of the free right ventricular wall (8). Additionally, it has been demonstrated that left ventricular diastolic dysfunction and elevated mortality are independently correlated with epicardial fat volume (EFV) (13). Current studies suggest that EAT may be associated with poor cardiovascular outcomes in patients with SSc and cardiovascular comorbidities (11–13).

Our aim in this study was to investigate the relationship between epicardial adipose tissue thickness and all-cause mortality at the end of a 10-year follow-up in patients with systemic sclerosis.

## 2 Methods

This is a retrospective observational cohort study conducted among patients diagnosed with systemic sclerosis (SSc) who were followed up in the Rheumatology and Cardiology outpatient clinics of the Health Sciences University Gazi Yaşargil Training and Research Hospital between January 2013 and December 2015. Sixty people diagnosed with SSc who were followed up in our hospital were included in the study. Participants were divided into two groups based on survival status at the end of follow-up: those who died (30 patients) and those who survived (30 patients). SSc was diagnosed according to the 2013 American College of Rheumatology/European League Against Rheumatism (ACR/EULAR) classification criteria. To minimize confounding factors, patients with known coronary artery disease, severe valvular disease, low left ventricular ejection fraction (less than 50%), unrelated chronic lung conditions, or concurrent autoimmune diseases were excluded from the study.

### 2.1 Data collection

Baseline clinical data were obtained from hospital records through patient interviews, chart reviews, and laboratory reports. Information recorded included age, sex, age at symptom onset and diagnosis, SSc subtype (limited, diffuse, sinus scleroderma, or overlap syndromes), and disease duration. Functional capacity was assessed using the New York Heart Association (NYHA) classification. The study protocol was approved by the Ethics Committee of the Gazi Yaşargil Training and Research Hospital of the University of Health Sciences (430-03/10/2025). The study was conducted in accordance with the updated principles of the 2013 Helsinki Declaration. Routine laboratory tests included measurement of antinuclear antibody (ANA) titers, and lung function was assessed using forced vital capacity (FVC). Skin involvement was assessed using the modified Rodnan skin score (mRSS). The presence of comorbidities such as hypertension and diabetes was also included in the records.

### 2.2 Echocardiographic evaluation and measurement of epicardial adipose tissue

All participants underwent transthoracic echocardiography by trained cardiologists blinded to patient outcomes. A standardized imaging protocol was used for consistency. Epicardial fat was assessed from the parasternal long-axis view and measured from the right ventricular free wall at end-systole. Measurements were averaged across three cardiac cycles to ensure reliability.

Right ventricular functional assessment included tricuspid annular plane systolic excretion (TAPSE), right ventricular myocardial performance index (MPI), estimated mean pulmonary artery pressure (PAP) derived from tricuspid regurgitation velocity, and aortic expansion velocity (APV), an indirect marker of diastolic function.

In a subset of patients, 24-h Holter monitoring was used to assess heart rate variability (HRV), including parameters such as the standard deviation of the normal-to-normal intervals (SDNN), the standard deviation of the mean of the normal-to-normal intervals across all 5-min segments (SDANN), and the root mean square difference between consecutive normal heartbeats (RMSSD). Echocardiographic and Holter analyses were performed by two independent investigators blinded to patient outcomes. Inter-measurement agreement was assessed by repeating measurements in 10 randomly selected patients.

### 2.3 Outcome definition and monitoring

The primary endpoint of our study was defined as all-cause mortality. Survival data were collected from institutional medical records and verified by direct contact with patients' families when necessary. The timing of death and duration of follow-up were recorded for all participants. Patients who experienced no events were censored at their last known follow-up visit.

## 3 Statistical analysis

SPSS version 26.0 for Windows (Armonk, NY, USA: IBM Corporation) was used to analyze the data. Histograms and the Shapiro–Wilks and Kolmogorov–Smirnov tests were employed to evaluate the normal distribution of continuous variables. The mean ± standard deviation (SD) is used to represent continuous variables having a normal distribution, while the median (IQR) is used to represent those with non-normal distributions. For continuous variables having a normal distribution, the Mann–Whitney *U* test was employed, and for non-normal distributions, the student's *t*-test. Categorical variables were compared using the chi-square test, also known as Fisher's exact test, and the results were presented as percentages. All-cause mortality predictors were examined using univariate and multivariate Cox regression models. Multivariate analysis comprised patients whose univariate *p*-value was less than 0.2. To illustrate the connection between cumulative events and HALP scores, ROC curve studies were conducted. The ROC curve study was based on the Youden index. A *p*-value of less than 0.05 indicated that a variable was significant.

## 4 Results

Baseline clinical characteristics of the study population are presented in Table 1, and regression analyses are summarized in Tables 2 and 3. A total of 60 patients diagnosed with systemic sclerosis were included in the study; 30 died (non-survivor group) and 30 were alive (survivor) at follow-up. Mean epicardial adipose tissue thickness (EATT) was significantly higher in the non-survivor group compared to the survivors (5.8 ± 0.7 mm vs. 7.1 ± 0.7 mm, *p* < 0.001). Similarly, pulmonary artery pressure was higher in the non-survivor group [35 (22) mmHg vs. 28 (10) mmHg, *p* = 0.014], and tricuspid annular plane systolic excursion (TAPSE) was significantly lower (23.6 ± 9.7 mm vs. 17.2 ± 10.5 mm, *p* = 0.017).

**Table 1:** Baseline characteristics of total population.

Parameters	Non-survivors (*n* = 30)	Survivors (*n* = 30)	Total (*n* = 60)	*p* value
Female gender, *n* (%)	28 (93.3)	30 (100)	58 (96.7)	0.150
Age (years)	48.4 ± 11.5	45.3 ± 10.3	46.9 ± 10.9	0.275
NYHA class	2.3 ± 0.8	1.9 ± 0.6	2.1 ± 0.7	0.067
Epicardial adipose tissue (mm)	7.1 ± 0.7	5.8 ± 0.7	6.4 ± 1	**<0.001**
Aortic propagation velocity (cm/s) Pulmonary artery pressure	50.2 ± 8.6	56.5 ± 7.3	53.3 ± 8.5	**0.003**
Mean pulmonary arterial pressure (mmHg)-IQR	35 (22)	28 (10)	32 (16)	**0.014**
TAPSE (mm)	17.2 ± 10.5	23.6 ± 9.7	20.4 ± 10.5	**0.017**
SDNN (msc)	94 ± 43	88 ± 19	93 ± 34	0.102
SDANN (msc)	82 (50)	77.1 ± 18.7	80 ± 32	0.124
RMSDD (msc)-IQR	40 (63)	31 (13)	33 (21)	**0.046**
Mean heart rate (bpm)	81.1 ± 10.8	81.1 ± 7.9	81 ± 9.4	0.989
MPIx100 (Right ventricle)	39 ± 17.4	30.9 ± 13.8	35 ± 16	**0.050**
Age at first diagnosis (years)	39.8 ± 11.5	36.7 ± 12	38.7 ± 11.2	0.231
Age at onset of first clinical sign (years)	42.8 ± 12.9	38.9 ± 12.1	40.9 ± 12.5	0.306
FVC (%)	73.1 ± 23.1	80.5 ± 16.8	76.8 ± 20.4	0.165
ANA (IU/mL)	6 ± 4.1	3.7 ± 1.8	4.8 ± 3.3	**0.009**
Left atrial diamete (mm)	48.7 ± 10.3	45 ± 11.4	46.9 ± 10.9	0.184
Rodnan score (mRSS)-IQR	11 (16)	11 (6.5)	11 (9)	0.410
Hypertension, *n* (%)	6 (20)	7 (23.3)	13 (21.7)	0.754
Diabetes mellitus, *n* (%)	5 (16.7)	3 (10)	8 (13.3)	0.448
Smoking, *n* (%)	2 (6.7)	5 (16.7)	7 (11.7)	0.228
Palpitation, *n* (%)	18 (60)	9 (30)	27 (45)	**0.020**
Dyspnea, *n* (%)	24 (80)	18 (60)	42 (70)	0.091
Angina, *n* (%)	11 (36.7)	6 (20)	17 (28.3)	0.152
Hemorrhagic cystit, *n* (%)	16 (53.3)	11 (36.7)	27 (45)	0.194
Family history of Scleroderma, *n* (%)	25 (83.3)	17 (56.7)	42 (70)	**0.024**
Disease type				**0.014**
Diffuse Cutaneous Systemic Sclerosis, *n* (%)	8 (26.7)	2 (6.7)	10 (16.7)	
Limited Cutaneous Systemic Sclerosis, *n* (%)	16 (53.3)	23 (76.7)	39 (65)	
SSc sine scleroderma, *n* (%)	1 (3.3)	0 (0)	1 (1.7)	
SSc with Secondary Sjögren's Syndrome, *n* (%)	4 (13.3)	0 (0)	4 (6.7)	
Systemic Sclerosis-Overlap with SLE, *n* (%)	1 (3.3)	5 (16.7)	6 (10)	

NYHA, New York Heart Association; TAPSE, tricuspid annular plane systolic excursion; msc, millisecond; mRSS, modified Rodnan skin score; SDNN, standard deviation of NN intervals; SDANN, standard deviation of average NN intervals; RRMSDD, Root mean square of successive differences; PNN50, percentage of NN intervals differing by more than 50 ms; MPI, myocardial performance index; SLE, systemic lupus erythematosus; SSc, systemic sclerosis; FVC, forced vital capacity; ANA, antinuclear antibody.

Bold values indicate statistically significant results (*p* < 0.05).

**Table 2:** Univariable and multivariable Cox regression analyses for all-cause mortality.

Parameters	Univariable		Multivariable	
	H.R. (95% CI)	*P* value	H.R. (95% CI)	*P* value
Epicardial adipose tissue	1.077 (1.036–1.121)	**<0.001**	1.120 (1.050–1.195)	**<0.001**
Diabetes mellitus	2.512 (0.926–6.817)	0.071	1.751 (0.418–7.339)	0.443
Mean pulmonary arterial pressure	1.015 (0.998–1.032)	0.078	0.995 (0.961–1.031)	0.797
Tricuspid annular plane systolic excursion	0.997 (0.994–1.001)	0.121	1.001 (0.997–1.006)	0.568
Aortic propagation velocity	0.953 (0.912–0.995)	**0.030**	0.979 (0.906–1.058)	0.589
Right ventricular MPI	1.020 (0.996–1.045)	0.106	0.980 (0.947–1.015)	0.258
Age at first diagnosis	1.024 (0.996–1.053)	0.099	0.915 (0.805–1.040)	0.175
Age at first clinical signs	1.026 (0.995–1.058)	0.097	1.135 (0.982–1.312)	0.087
Age	1.016 (0.982–1.051)	0.370		
Female gender	1.650 (0.390–6.979)	0.496		
Mean heart rate	0.978 (0.941–1.016)	0.250		
Hypertension	0.724 (0.296–1.775)	0.481		
Smoking	0.604 (0.143–2.550)	0.493		

Bold values indicate statistically significant results (*p* < 0.05).

**Table 3:** Pearson correlation analysis of epicardial adipose tissue and right ventricular functions.

Parameters	Pearson correlation coefficient	*P* value
Age	0.090	0.493
Aortic propagation velocity	−0.509	**<0.001**
Mean pulmonary arterial pressure	0.345	**0.007**
Tricuspid annular plane systolic excursion	−0.591	**<0.001**
Mean heart rate	−0.106	0.421
Myocardial performance index of the right ventricle	0.368	**0.004**
Age at first diagnosis	0.038	0.771
Age at first clinical signs	0.095	0.470

Bold values indicate statistically significant results (*p* < 0.05).

### 4.1 Correlation between epicardial adipose tissue and cardiac parameters

**Figure 1:**
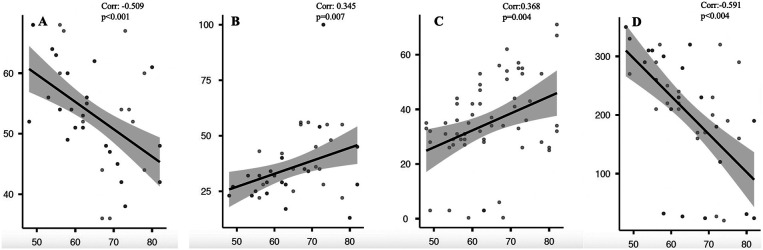
Correlation of EATT and APV **(A)**, mPAP **(B)**, MPI **(C)**, TAPSE **(D)**.

Pearson correlation analysis showed significant associations between EATT and multiple right heart function parameters. EATT showed a strong negative correlation with aortic propagation velocity (APV) (*r* = –0.509, *p* < 0.001; Figure 1A) and TAPSE (*r* = –0.591, *p* < 0.001; Figure 1D), indicating that increased adipose tissue is associated with right ventricular diastolic and systolic dysfunction. Conversely, a positive correlation was found between EATT and both mean pulmonary artery pressure (mPAP) (*r* = 0.345, *p* = 0.007; Figure 1B) and myocardial performance index (MPI) (*r* = 0.368, *p* = 0.004; Figure 1C), further highlighting the link between increased adipose accumulation and right ventricular dysfunction.

### 4.2 Prognostic value of epicardial adipose tissue thickness

**Figure 2:**
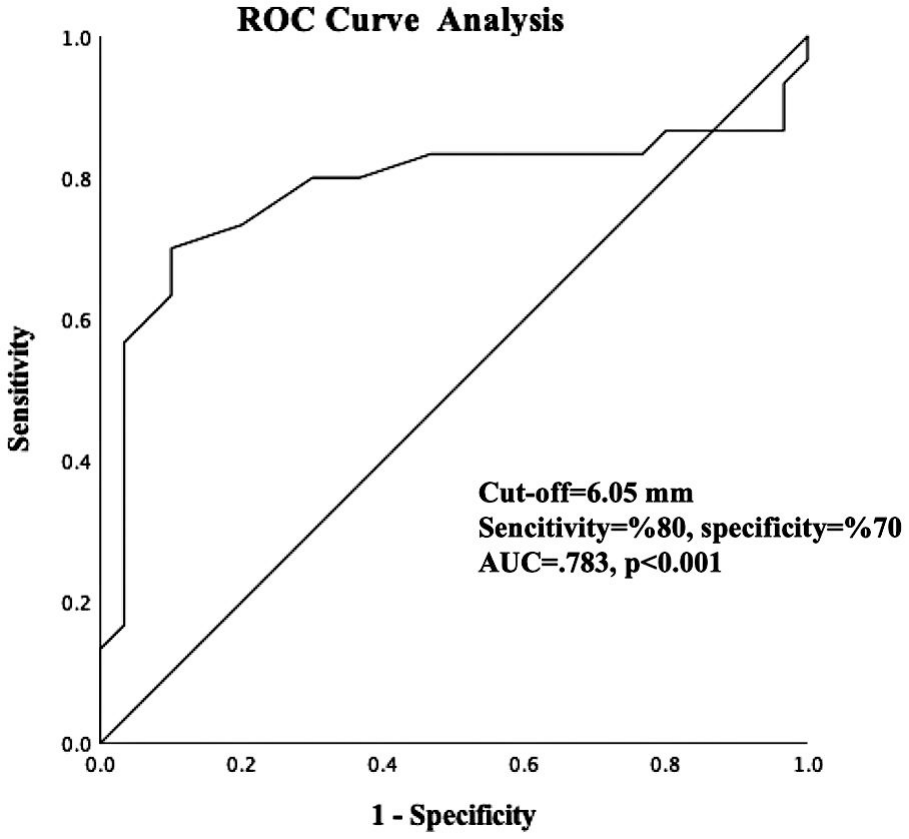
Forest-Plot graph of multivariable Cox regression analyses.

Univariable Cox regression analysis found EATT to be a significant predictor of all-cause mortality (HR: 1.077; 95% CI: 1.036–1.121; *p* < 0.001). EATT was also associated with mortality after adjustment for diabetes, mean PAP, TAPSE, aortic propagation velocity, right ventricular MPI. However, the absence of key prognostic variables (ILD and antibody profile) in the model does not allow us to conclude on an independent role. None of the other clinical or echocardiographic variables, including age, TAPSE, MPI, or mPAP, remained statistically significant in the adjusted model. The results of the multivariable Cox regression analysis are illustrated in Figure 2.

### 4.3 Diagnostic performance of EAT

**Figure 3:**
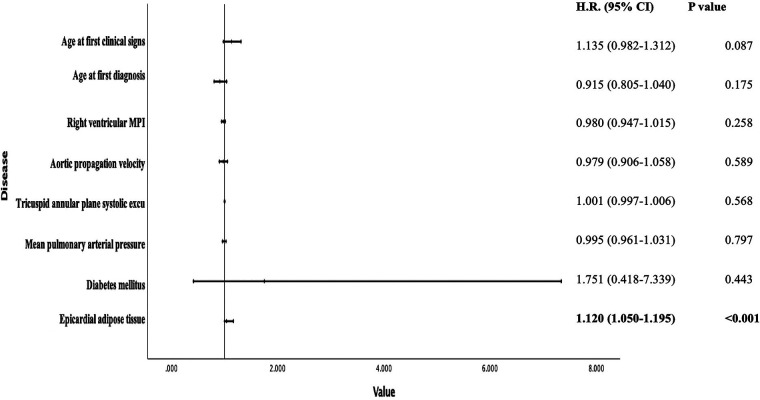
Diagnostic performance of EAT thickness for All-cause mortality: ROC curve illustration.

Receiver operating characteristic (ROC) curve analysis revealed that an EATT cutoff value of 6.05 mm could effectively discriminate the risk of mortality with an area under the curve (AUC) of 0.783 (*p* < 0.001), achieving 80% sensitivity and 70% specificity (Figure 3).

## 5 Discussion

Based on the findings of our study, we found that epicardial fat tissue thickness could predict mortality in patients with systemic sclerosis. Additionally, EATT was associated with mortality when we included various parameters that could be related to mortality in our model analysis. Furthermore, ROC curve analyses indicated that an EATT value of 6 mm may be a significant cutoff in patients with systemic sclerosis. The correlation between EATT and TAPSE, right ventricular MPI, and APV, which are markers of right heart function, indicated that EATT may be an indirect indicator of right heart function.

Epicardial adipose tissue is recognized as a metabolically active organ capable of secreting pro-inflammatory cytokines and adipokines that may contribute to endothelial dysfunction, microvascular injury, and myocardial fibrosis. In systemic sclerosis, where immune activation and vascular damage play a central role, EAT may represent not only a structural marker but also a potential inflammatory mediator. Although inflammatory biomarkers were not available in our study, the observed association between EATT and right ventricular dysfunction supports the hypothesis that EAT-related inflammatory activity may contribute to adverse cardiovascular remodeling. Future prospective studies incorporating systemic inflammatory markers are needed to clarify this potential mechanistic link (11, 12).

Previous studies showing the relationship between EAT thickness and poor cardiovascular outcomes are available in the literature. Our study is largely similar to the results of previous studies. A recent study by Yang et al. showed that high epicardial fat volume was an independent risk factor for MACE in patients with normal ejection fraction and suspected or known coronary artery disease (CAD) after adjusting for the traditional risk factors of coronary artery disease (CAD) and MPI (13). Additionally, Huang et al. found that elevated epicardial fat volume levels were independently associated with the risk of MACE in SSc patients and were predictive of poorer prognostic outcomes in SSc patients, even those unaffected by PAH. They argued that the use of epicardial fat volume may be more useful in predicting the incidence of MACE than traditional risk factors (8). In another previous study, Mohamed AAO and colleagues showed that EAT levels may be significantly associated with the risk of cardiac dysfunction and death in many systemic inflammatory diseases, including systemic sclerosis (14). Similar to these studies, we showed that EAT thickness, although not epicardial fat volume, was associated with 10-year all-cause mortality.

Increased epicardial fat volume in patients with systemic sclerosis is associated with the presence and severity of SSc, independent of cardiovascular risk factors and interstitial lung disease (15). The effect of increased epicardial fat tissue on cardiac functions, especially in the right ventricle, has been tested in some previous studies. Lu et al. showed that epicardial fat tissue exhibits distinct electrophysiological effects on the right ventricular outflow tract, which is prone to ventricular arrhythmia induction, and this may play a role in ventricular arrhythmogenesis related to the pathogenesis of lipotoxicity (16). Song et al. showed in their research that right ventricular systolic function and early diastolic function were impaired in patients with Type 2 DM, and this was associated with thickening of the EAT (17). In a study by Schulz et al., they demonstrated that signs of diastolic functional insufficiency and adverse structural remodeling were more pronounced in patients with diastolic dysfunction and elevated EAT. Despite similar morphological features, patients with elevated EAT exhibited significant cardiac functional impairment, particularly in the atria (18). EATT is expected in patients with systemic sclerosis. Increased EATT is also associated with adverse cardiac outcomes. Our study's results are consistent with previous studies. In our study, we found a positive correlation between EAT and mPAP and right ventricular MPI, and a negative correlation between TAPSE and APV.

In addition to these results, our research provides additional evidence for the predictive significance of EATT in systemic sclerosis patients. Among the 60 patients in the study, the non-survivor group had significantly higher mean pulmonary artery pressure [35 (22) mmHg vs. 28 (10) mmHg, *p* = 0.014], lower TAPSE (17.2 ± 10.5 mm vs. 23.6 ± 9.7 mm, *p* = 0.017), and significantly higher EATT values (7.1 ± 0.7 mm vs. 5.8 ± 0.7 mm, *p* < 0.001). These findings are in line with other research showing a link between elevated EAT and negative consequences and right ventricular dysfunction. Additionally, EATT demonstrated a positive association with both mPAP and MPI, indicators of right heart strain, and a substantial inverse correlation with TAPSE and APV. Crucially, even after controlling for pulmonary hypertension and diabetes, multivariable Cox regression analysis revealed that EATT was an independent predictor of all-cause death (HR: 1.120; 95% CI: 1.050–1.195; *p* < 0.001). ROC analysis results highlight the clinical utility of EATT as a noninvasive and practical tool for risk stratification in patients with systemic sclerosis, reinforcing prior observations in the literature regarding its role in predicting mortality and right heart dysfunction.

Microvascular injury is a central component of systemic sclerosis pathogenesis and plays a critical role in the development of pulmonary hypertension and right ventricular dysfunction. Nailfold capillaroscopy provides important insight into the degree of microangiopathy and disease progression. Although capillaroscopic data were not available in our cohort, it is plausible that advanced microvascular damage may coexist with increased epicardial adipose tissue and contribute to adverse cardiovascular remodeling. Future prospective studies integrating capillaroscopic patterns with cardiac imaging parameters may further clarify this relationship.

However, the effects of epicardial adipose tissue (EAT) are not limited to the cardiovascular system. Indeed, the literature has shown that EAT may also play a role in non-cardiovascular processes such as lung fibrosis through adipokine release and local inflammatory effects (19, 20). These findings suggest that the effects of EAT on mortality may be multifaceted and cannot be explained solely by vascular pathways. However, due to data limitations, we cannot draw any conclusions on this topic based on the results of our study.

We believe that the findings of our study have significant prognostic value in patients with systemic sclerosis who are at such a high risk of mortality. We believe that the clinical use of epicardial adipose tissue, which can be easily and noninvasively measured, can aid clinicians in patient management.

### 5.1 Limitations

As with many studies, our study has several limitations. Our study is a single-center, retrospective study. The limited number of patients included and the small size of the study make generalization difficult. Furthermore, we were unable to perform G-Power analysis to determine the sample size because all patients we followed were within this scope. Although we have 10-year mortality data, some data that could potentially affect the results, such as patient medication use and causes of death, are missing. Another limitation of our study is that we measured two-dimensional EAT thickness. Adding epicardial fat volume or epicardial fat volume index to the findings could have yielded more reliable results. A significant limitation of our analysis is that we could not include all known prognostic markers, such as interstitial lung disease (ILD). While some data in patient records, such as FVC levels and chest CT reports, could indicate the presence of ILD, this information was not considered reliable for variable identification due to its retrospective nature and lack of standardization, and was therefore excluded from the analysis. This situation highlights the need for caution in interpreting our results, particularly given the significant role of ILD in determining mortality. Systematically determining the presence of ILD in prospective studies will be important to improve model accuracy. Furthermore, patients were not adequately characterized. Important parameters such as BMI, metabolic status, and antibody positivity (Scl-70, anti-centromere, and anti-RNA pol III) were unavailable due to the retrospective design. These shortcomings may have affected the study results. In our study, data on causes of death were limited; particularly in deaths occurring at home, it was not possible to differentiate between cardiovascular and non-cardiovascular causes, as the cause was not clearly stated in official records. Causes of mortality could not be classified in a detailed and standardized manner. Due to the retrospective design and limited death records in some patients, we could not definitively determine whether deaths were due to atherosclerotic heart disease, pulmonary arterial hypertension, interstitial lung disease, or other non-cardiovascular causes. Therefore, it is not possible to say that epicardial adipose tissue thickness is specifically associated with atherosclerotic cardiac mortality. Our findings should only be interpreted in terms of all-cause mortality, and this requires careful evaluation of the results. Nail bed capillary patterns were not systematically documented in medical records due to the retrospective design and therefore could not be analyzed. This limits our ability to examine the relationship between microvascular severity and cardiovascular outcomes. Systemic inflammatory biomarkers such as CRP, ESR, or BNP were not consistently available due to the retrospective design and therefore were not included in the analysis. Given the inflammatory nature of epicardial adipose tissue, the absence of these markers limits our ability to investigate potential mechanistic links between inflammation, EAT, and cardiovascular risk. Prospective studies including standardized inflammation assessments are needed. EATT may also be a predictor of non-cardiovascular deaths, but we cannot make any assessment on this due to a lack of data.

## 6 Conclusion

We found that the findings of our study have significant prognostic value in patients with systemic sclerosis who are at such a high risk of mortality. The clinical application of EATT, which can be easily and noninvasively measured, can aid clinicians in patient management. Consider EATT as a warning sign, and closely monitoring it in this patient group may have positive implications for patient management.

## Data Availability

The original contributions presented in the study are included in the article/Supplementary Material, further inquiries can be directed to the corresponding author.
